# The Influence of Mimicry on the Reduction of Infra-Humanization

**DOI:** 10.3389/fpsyg.2016.00975

**Published:** 2016-06-28

**Authors:** Anna Szuster, Agnieszka Wojnarowska

**Affiliations:** Faculty of Psychology, Warsaw UniversityWarsaw, Poland

**Keywords:** mimicry, imitating, infra-humanization, emotions, attitudes, embodiment

## Abstract

The paper investigates the role of mimicry in the reduction of infra-humanization. Mimicry as an automatic imitation of a partner’s behavior (this is known as “social glue”) connects people (Miles et al., 2010). Empirical findings have confirmed that mimicry leads to favorable treatment of the mimicker (Van Baaren et al., 2003). The mechanism is reciprocal: the mimicker is more positively inclined towards the person mimicked (Chartrand and Bargh, 1999). Mimicry increases the sense of interpersonal closeness, reciprocal similarity, and facilitates the flow of interaction and helping behavior (Stel et al., 2008). At the same time, results point to a spontaneous inhibition of mimicry in the contact with members of negatively stereotyped groups (Bourgeois and Hess, 2008). So than, there are reasons to believe that purposeful activation of mimicry may reduce manifestations of negative attitudes towards others, i.e., infra-humanization. It was expected that imitating a model shown on video would lower the level of infra-humanization, i.e., perceiving another individual as less capable than the Self of experiencing exclusively human (secondary) emotions. The study sample consisted of 117 female students. The study followed an experimental design and was conducted individually. It employed questionnaires and a purpose-made video recording showing a model whose facial expressions were to be mimicked by the participants. The results confirmed the predictions. Mimicking facial expressions reduced the level of infra-humanization compared to the no mimicking and control conditions [*F*(2,114) = 3.39, *p* = 0.037, *η*^2^ = 0.06].

## Introduction

The purpose of the study was to explore factors that mitigate the negative aspects of intergroup attitudes reflected in infra-humanization. The focus was on the area framed by embodiment theories, in particular on the regulatory function of mimicry. Our research was inspired by numerous reports indicating the mediating role of mimicry in enhancing prosocial behavior. The main starting point was research conducted by Stel et al. (2008), which showed that donations to a charity increased when subjects mimicked a representative of that charity. The effect turned out to be non-specific: it was still observed when the mimicked actor was not associated with the charity receiving donations. This proves that mimicry can modify social attitudes by enhancing prosocial attitudes observed in the model. The mechanism is primary and automatic. In addition, the beneficial effects of prosociality are augmented even if the person being mimicked is not associated with the actual beneficiary of a charitable act. Due to the basic nature of the regulatory function of mimicry, it can be expected to affect other aspects of social behavior as well. Its inhibitory effect on negative attitudes, including infra-humanization, appears to have important theoretical and practical implications.

### Infra-Humanization

A number of studies have shown that people tend to perceive the quality of being human as an essential feature of their in-group, while denying that same quality to the members of an out-group. This is reflected in the asymmetrical attribution of emotions recognized as typically human (secondary), and emotions experienced by people and animals alike (primary). While the intensity of experiencing primary emotions is not a differentiator between in-group and out-group, secondary emotions are more readily attributed to members of one’s ingroup (Leyens et al., 2001). Thus, the quality of being human is not something permanent and inalienable, but rather a dynamic dimension of comparisons.

The phenomenon of infra-humanization has been shown to be universal (Demoulin et al., 2009). Rather than denying other people’s humanity, it involves relative differentiation in ascribing uniquely human traits to one’s fellows and strangers. An out-group can still be seen as human, only to a lesser degree than the in-group. Infra-humanization has been shown to be independent of negative attitudes toward strangers: it affects both positive and negative emotions. It emerges between groups even in the absence of objective reasons [e.g., lack of conflicting interests between French and Spanish students or between students from continental Spain and the Canary Islands, as in the research conducted by Leyens et al. (2001)]. Moreover, it is not exclusive to relations with the least favorably perceived out-groups: the best-liked ones also tend to be seen as less human (Vaes and Paladino, 2010). The sole prerequisite for its emergence is an act of categorization.

Infra-humanization is an implicit, automatic process. People are not aware of applying inconsistent standards when assessing the emotional functioning of others. The effect is revealed with the use of implicit cognition measures, such as the Implicit Association Test (IAT). The time of categorization of secondary emotions in the “US” category was found to be shorter than in the case of the “OTHERS” category (Leyens et al., 2001; Boccato et al., 2007).

Although traditionally the phenomenon of infra-humanization has been seen as an aspect of intergroup essentialism, i.e., perceiving others as qualitatively different from one’s own group, results obtained by Haslam (2006) showed that it also operates on the individual level. Unfamiliar individuals not perceived in terms of group affiliation were also seen as less capable of experiencing exclusively human emotions.

### Mimicry

Everything we do, including the way we perceive the world, is “filtered” through our physicality. The last decade witnessed a breakthrough in the understanding and recognition of the role of physical body in social cognition. In embodied cognition theories, the body is considered to be the key factor in the development of the mind (Clark, 1997; Barsalou, 1999), and sensory perception (especially visual) is at the center of attention. The “body to mind” approach describes the organism as an active system of cognition, registering external stimuli and their variations, and converting them into information. In addition, this cognitive system draws on its own resources of imagination and memory, along with inference processes. The key concepts of the embodied cognition paradigm include: (i) the metaphor of the mind as the product of structural coupling, in which cognitive processes result from a specific type of embodiment, environment, and action; (ii) the relational approach: cognition can be explained by the interrelations between the mind, body, and environment; (iii) belief in the superiority of directional, result-driven activities in real time over “calculability”; (iv) viewing cognition as an active construct grounded in embodied processes; and (v) the sensorimotor nature of cognitive representations (Clark, 1997). The last concept is of particular importance for the analysis of embodiment’s implications for social attitudes. The hypothesized modality of cognitive processes assumes that they are based on a certain “reproduction” of sensorimotor states. To “know” something is not merely to fashion an abstract structure (representation) in the mind. It necessarily involves a re-enactment of a given event through those sensory processes that were involved in the perception of the original stimulus (Niedenthal, 2007). Such modal cognitive representations may be activated by body movements that reproduce them. This type of sensorimotor reactivation is selective, dependent on the current significance of a given piece of information and focus of attention (Barsalou, 1999).

The embodied cognition concept finds support in the results of research on the regulatory role of mimicry, which suggest that observation of facial expressions provides information that facilitates both understanding and sending of messages. Perception of facial expressions is accompanied by the activation of muscle groups consistent with observed expressions (Dimberg and Thunberg, 1998). Studies confirm the prevalent tendency to spontaneously coordinate behavior, including facial expressions, with the partner of interaction (Cappella, 1997; Chartrand et al., 2005; Miles et al., 2010).

By activating peripheral mechanisms, the automatic capacity for mimicry supports perception and decoding of facial expressions. Adopting the appropriate facial expression alone may be sufficient to evoke a given emotion. Inhibiting facial expressions of joy or sadness by having participants hold a pencil in their mouth altered their ratings of how funny drawings were (Strack et al., 1988). In another research project (Duclos et al., 1989), participants were asked to tense specific muscles in a detailed instruction that made no mention of a corresponding emotion. There were four patterns: fear, sadness, anger, and disgust. A given facial expression was maintained for 6 s, after which participants rated the intensity of experiencing nine different emotions on a scale. Across all conditions, participants experienced the emotion that they mimicked earlier as stronger. Thus, the feedback loop facilitates the identification of content and positive/negative valence of emotion. The underlying neuronal structure involves mirror neurons, responsible for coding whole movement sequences. The effect of mimicry on attitudes is that of increased favoring of the mimickee (e.g., larger tips offered to bartenders mimicking speech and facial expression; Van Baaren et al., 2003). The mechanism is reciprocal – the mimicker is more positively inclined towards the person he mimics (Chartrand and Bargh, 1999; van Baaren et al., 2004; van der Velde et al., 2010). Some researchers have reported that mimicking stimulates interpersonal closeness as well as perceived similarity to the self, and enhances the flow of interaction (Bernieri and Rosenthal, 1991; Chartrand and Bargh, 1999; Bailenson and Yee, 2005; Stel et al., 2006). Mimicry is often referred to as “social glue,” which binds people together and creates harmonious relations (Lakin et al., 2003, p. 147).

The majority of studies focus on the prosociality of mimickers in order to prove that mimicry increases willingness to help, e.g., spontaneously pick up coins dropped “by accident”. Help was offered both to the mimickee and to bystanders. The people being mimicked were more likely to donate to charities (van Baaren et al., 2004). Further experiments (Stel et al., 2008) have demonstrated the same mechanism for mimickers. Participants were shown a short film in which a member of an animal rights charity (PETA) described activities aimed at helping animals. They were asked either to mimic or not to mimic. At the exit, participants could donate to PETA. Those in the mimicry condition donated more (1.06 EUR on average) than those in the no mimicry condition (0.3 EUR on average). The same relationship was found when the mimickee had no affiliation with the charity to which participants could donate. In our study we also found the effect of mimicry on the amount of donations for the construction of a clinic for pediatric coma patients. The effect was non-specific and unrelated to the object being mimicked, which was a young woman talking about her career (Szuster, 2012).

### The Current Research

A question that arises is whether the effect of mimicry is selective (Macrae and Johnston, 1998; Ashton-James et al., 2007; Stel et al., 2008), enhancing positive attitudes by boosting prosociality, as in the Stel study, or if it can also be effective in reducing negative attitudes, such as those resulting from infra-humanization?

A review of research suggests that activation of mimicry is generalized: it enhances prosocial behavior both within and outside of the context in which it was activated, as well as when the mimicked model presents a prosocial message and when the prosocial context is not activated. On the other hand, we know from empirical data that mimicry is inhibited in contacts with members of negatively stereotyped groups (Bourgeois and Hess, 2008). This phenomenon could shape low-level response habits that reinforce negative attitudes towards strangers. Thus, research findings suggest that mimicry is important in regulating both positive and negative attitudes, and may even constitute the embodiment basis of stereotypes. The automatic character of mimicry does not restrict its selectiveness, as its activation depends on the particular circumstances.

Consequently, there are reasons to believe that purposeful activation of mimicry, by triggering what is hypothesized to be a generalized change towards increased focus on other people (Macrae and Johnston, 1998; Ashton-James et al., 2007; Stel et al., 2008), may reduce manifestations of negative attitudes towards others, i.e., infra-humanization. Furthermore, both infra-humanization and mimicry are activated at a similar, automatic level. Based on this premise, mimicry can be expected to affect infra-humanization.

### Hypothesis

Situational activation of mimicry modifies the magnitude of the infra-humanization effect such that infra-humanization will be lower in the condition where facial expressions are mimicked compared to the no-mimicry condition and the control condition.

## Materials And Method

### Design and Participants

The study followed an experimental design and was conducted individually. One independent situational variable – mimicking, no mimicking of another person’s facial expressions, and the control condition – and one dependent variable – the level of infra-humanization – were operationalized. Female students of the University of Warsaw aged 19–24 non-working participated in the study (*n* = 117). The study only enrolled unemployed participants, since the employment vs. studying dimension was to be the criterion of categorization, which is the basis of the infra-humanization effect. In order to eliminate the effect of gender, the sample included only females. In terms of the manipulated variable (mimicking vs. no mimicking condition), the sample size in both experimental conditions was *n* = 40 and *n* = 37 in control condition. The Ethics Board of the Faculty of Psychology at the University of Warsaw approved the study, which was carried out in accordance with the Board’s recommendations.

### Conditions

The mimicking conditions were operationalized by verbal instructions asking participants to either mimic or not mimic facial expression of the person watched on screen. Participants were shown a purpose-made 5-min recording, showing a young woman (Caucasians) talking about her career in the big media company. The film featured a young person sitting on a chair, seen from her waist up and talking animatedly about her career in a big corporation. The person did not display vivid emotions. At times she would smile mentioning her successes. Also, with the mention of the long working hours, her slight tiredness would show. A resigned look at her face appeared when she mentioned the fact that she was made to take her vacation in the autumn, not in the summer; then, she would talk with interest about a new project. The person complained on the little time she had for herself or her social life. In her opinion the pay and the forthcoming promotion made up for these disadvantages. Summing up, she talked about the importance of this experience, which is not, however, meant for life. So she displayed quite different emotions. In the control condition, the video was played without any instructions. Compliance with instructions was monitored using the Face-Reader software.

### Operationalization of Infra-Humanization

The scale constructed by Leyens et al. (2001) was used. It lists 24 emotions, 12 of which are secondary and 12 primary, balanced in terms of valence (6 positive and 6 negative in each category). The task is to indicate on a 5-point scale below each emotion how often the participant experiences it in everyday life: from 1 meaning “never” to 5 meaning “very often”. Participants then completed the same questionnaire for the person in the recording. The original Spanish scale was standardized in Poland (Baran, 2007).

(1) *Examples of primary emotions*: fear and excitement.
(1) *Examples of secondary emotions*: hope and sense of guilt.

### Procedure

The study was conducted on an individual basis. Participants were told that the purpose of the study was to learn about the principles that govern perception of people’s emotional functioning. Then they were asked to complete the first part of the Leyens’ scale, in which they indicated the frequency of experiencing emotions themselves. Next, the recording was shown. Depending on the condition, it was preceded by the instruction to mimic or to refrain from mimicking the person on screen. No instructions were given in the control condition prior to video presentation. Participants were only informed (as in the other conditions) that the video is shown to give an idea of how people functioned in large corporations, where work takes a significant portion of their lives. Finally, participants completed the second part of the Leyens’ scale, this time with reference to the person in the recording.

## Results

In order to test the hypothesis regarding the impact of mimicry upon attribution of the valence of primary and secondary as well as positive and negative emotions to the self and the mimicked person (watched on screen) a 2 (object: the self/she) × 2 (emotion type: primary/secondary) × 2 (valence: positive/negative) × 3 (conditions: mimicry/no mimicry/control) ANOVA was calculated, with the first three factors varying within subjects, and the last one between subjects.

Two main effects were revealed: object type and emotion type. Object type (the self vs. the other) was found to significantly differentiate the valence of attributed emotions [*F*(1,114) = 76.46, *p <* 0.001, *η*^2^ = 0.40], a higher valence of all emotions was attributed to the self (*M* = 3.23) than the other (*M* = 2.87). Types of attributed emotions turned out to be significantly differentiated [*F*(1,114) = 24.06, *p <* 0.001, *η*^2^ = 0.17]. A markedly higher number of primary emotions was attributed (*M* = 3.13) compared with the secondary ones (*M* = 2.94).

**FIGURE 1:**
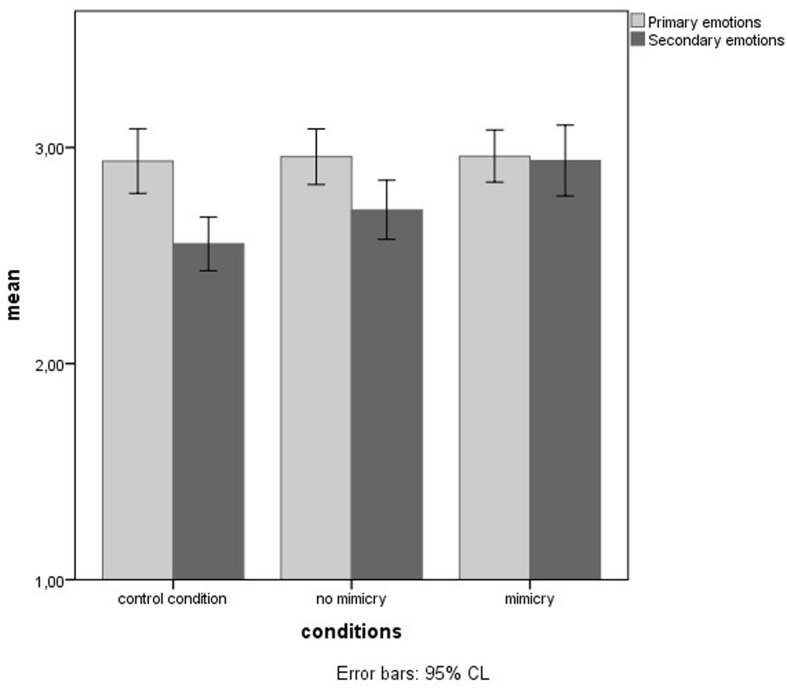
**Average valence of primary and secondary emotion attributed to the other in mimicry, no mimicry and control conditions**.

Most importantly, as predicted, the results show a significant interaction of conditions, type of emotion and object [*F*(2,114) = 3.39, *p* = 0.037, *η*^2^ = 0.06]. Participants in mimicry conditions attributed a higher valence of secondary emotions to the other (*M* = 2.94) compared to no mimicry conditions (*M* = 2.71), *p* = 0.034, and to the control condition (*M* = 2.55), *p* = 0.001. No significant difference between the no mimicry and control condition was found. There was also no significant difference in the attribution of primary emotions to the other between conditions. No other interaction effects were found (**Figure 1**).

The results confirm the hypothesis: the mimicry condition indeed modified the magnitude of infra-humanization.

An additional analysis taking into account the negative/positive valence of emotion was also conducted. Its purpose was to find out if categorization, which is the basis of infra-humanization, also generates effects reflecting favoritism, i.e., seeing the other as experiencing positive emotions to a lesser extent than the self. The analysis confirmed the specificity of infra-humanization and its distinctiveness from other intergroup phenomena: the interaction of conditions, valence of emotion and type of object, was not significant [*F*(2,114) = 0.74, ns]. All presented results shows that mimicry has a specific and selective influence that limits the effect of infra-humanization.

## Discussion

The present study assessed the effect of mimicking facial expressions on the level of infra-humanization in interpersonal relations.

The results support the hypothesis that mimicry reduces infra-humanization at the individual level.

Predictions relating to the regulatory function of mimicry have also been confirmed with respect to negative attitudes. This finding is consistent with the claims that mimicry tends to be completely or partially inhibited in interactions with members of negatively stereotyped out-groups (Bourgeois and Hess, 2008). While the latter effect is involuntary, automatic and occurs in a natural context, in the present study the inhibition and activation of mimicry were both intentional. Still, despite the somewhat compromised ecological validity, mimicry proved effective, confirming the primary and universal nature of the phenomenon.

Mimicry reflects the ability to construct embodied stimulation. As such, it plays a vital role in the perception and understanding of emotions (Niedenthal et al., 2001). The present findings demonstrate that, by reducing infra-humanization, mimicry is relevant to the quality of social functioning.

Any type of social interaction involves mirroring. This process is reflected, for example, in the modification of the self–other overlap (Aron et al., 2005). Mimicry, like the exchange of simple touches, serves to bridge psychological distance to others (Smith and Semin, 2004), while increasing their accessibility. This in turn can facilitate the processing of information in categories similar to those that are activated when the image of oneself and one’s group is constructed, along with the dimensions relating to emotional functioning. It has been suggested (Rizzolatti and Luppino, 2001; de Vignemont and Singer, 2006) that the neural basis of mimicking effects is the activation of brain regions responsible for empathy, mirror neurons in particular.

Research on the inhibition of infra-humanization tends to focus on the importance of cognitive factors. The effect can be reduced or even completely blocked in the condition with freely available cognitive resources and intentional activity (Leyens et al., 2007). Another factor is contact (Brown et al., 2007), including imagined contact (Vezzali et al., 2012). With time, contact facilitates the recognition of secondary emotions in members of out-groups. Our results indicate the presence of a factor that may mediate contact on a more basic, physical level. They can also be interpreted from the perspective of the pro-social mind-set proposed by Stel et al. (2006, 2008), which enhances positive attitudes and may at the same time reduce negative ones.

Our findings also suggest a selective effect of mimicry, since the effect is not related to the negative/positive valence of emotions, but only concerns their primary/secondary nature. This sets infra-humanization apart from other intergroup phenomena, such as favoritism and discrimination, and confirms its uniqueness. The underlying mechanisms responsible for their inhibition are distinct.

The lack of difference between the no mimicry and control conditions in terms of the attribution of secondary emotions to the other merits a more detailed discussion. This finding may suggest that, despite the automatic nature of mimicry, its intensity can be modified depending on circumstances or type of object. In other words, although the mimicry process is triggered automatically in the context of social interaction, its intensity varies. This interpretation finds further support in the findings from research on the underlying neuronal mechanisms of empathy. Authors used functional MRI (fMRI) while subjects were either imitating or simply observing emotional facial expressions. Imitations and observations of emotions activated a largely similar network of brain areas. Within this network, activity was greater during imitation compared to when emotions were merely observed (Carr et al., 2003). In our studies, the recording of facial muscle activity using Face Reader was intended to verify the effectiveness of manipulation. As a result, it could have provided information on the level of mimicry activation also in the control condition. Unfortunately, the analysis of recorded measurements was inconclusive due to poor recording conditions (lighting, head movements). Still, we can assume that participants, being aware of the measurement, attempted to comply with the instruction. This, in turn, suggests the emobodiment underpinnings of infra-humanization.

### Limitations

There remains some doubts whether the source of infra-humanization limitation was the intention (induced instructions) or the imitation *per se*. In the cited the results of study (Carr et al., 2003) used fMRI to indicate that imitations and observations of emotional facial expressions activated a largely similar network of brain areas. Within this network the activity during imitation was greater than the one which occurred when emotions were merely observed. The increased activity in the imitation condition is probably connected with the high road activation (LeDoux, 1996). In this case mimicry is the result of conscious intention entailing a reflective mode of processing (Petty and Cacioppo, 1979). Nevertheless, these results show that even if the subjects, acting in the experimental conditions, did not imitate but merely processed the instruction and watched the model, it would be accompanied by the activity of very similar neuronal networks. These data somewhat narrow the interpretation, according to which it is the mere intention (*per se*) that accounts for the effect, as it is accompanied by observation of the model which activates neuronal networks. At the same time the data confirm a fundamental regulatory role of the embodiment mechanisms. At this point a distinctly selective nature of mimicry impact should be stressed. It does not concern the sign-differentiated emotions, but is limited to specifically human, secondary emotions, that status of which is not consciously discernible by people (Leyens, 2000).

A limitation of the study, is also its non-ecological character. This puts into question the actual cause of the effect: was it mimicry that triggered the neuronal mechanism of empathy or the content of the instruction. The lack of differences between the experimental and control conditions is not conclusive in determining which level – physiological or cognitive – modified the attribution of emotions. A successful measurement of facial muscle activation in each condition would go a long way towards resolving this dilemma. This, then, is the issue that requires further empirical testing. Our findings are a starting point for further research on the embodiment basis of infra-humanization.

### Application

The present findings can serve as a beacon for developing interventions based on those basic, “embodied” processes, as well as elements of non-verbal communication, such as eye contact, physical distance and active listening to mitigate negative consequences of categorizing others, especially among young people. The alarming prevalence of cyberbullying among teenagers is largely due to the fact that online there are no non-verbal cues serving as natural inhibitors of aggression (Barlińska et al., 2013). The results of our other studies have confirmed the significance of exposure of a universal stimulus, the human face with the expression of a primary emotion in the form of fear, in the reduction of negative behavior on the Internet (Szuster et al., 2016). Mere exposure of a face activating the embodiment mechanisms of empathy significantly limits cyberbystander reinforcing of bullying behavior. The specific characteristics of adolescents make them particularly susceptible to interventions involving such embodiment-related factors. Educators and teachers should take interest in making them part of workshops and educational programs.

## Author Contributions

All authors listed, have made substantial, direct and intellectual contribution to the work, and approved it for publication.

## Conflict of Interest Statement

The authors declare that the research was conducted in the absence of any commercial or financial relationships that could be construed as a potential conflict of interest.
